# Prognostic factors for medical and productivity costs, and return to work after trauma

**DOI:** 10.1371/journal.pone.0230641

**Published:** 2020-03-25

**Authors:** Leonie de Munter, A. J. L. M. Geraerds, Mariska A. C. de Jongh, Marjolein van der Vlegel, Ewout W. Steyerberg, Juanita A. Haagsma, Suzanne Polinder

**Affiliations:** 1 Department Trauma TopCare, ETZ hospital (Elisabeth-TweeSteden Ziekenhuis), Tilburg, The Netherlands; 2 Department of Public Health, Erasmus University Medical Centre, Rotterdam, The Netherlands; 3 Brabant Trauma Registry, Network Emergency Care Brabant, Tilburg, The Netherlands; 4 Department of Biomedical Data Sciences, Leiden University Medical Center, Leiden, The Netherlands; University Hospital Zurich, SWITZERLAND

## Abstract

**Aim:**

The aim of this study was to determine prognostic factors for medical and productivity costs, and return to work (RTW) during the first two years after trauma in a clinical trauma population.

**Methods:**

This prospective multicentre observational study followed all adult trauma patients (≥18 years) admitted to a hospital in Noord-Brabant, the Netherlands from August 2015 through November 2016. Health care consumption, productivity loss and return to work were measured in questionnaires at 1 week, 1, 3, 6, 12 and 24 months after injury. Data was linked with hospital registries. Prognostic factors for medical costs and productivity costs were analysed with log-linked gamma generalized linear models. Prognostic factors for RTW were assessed with Cox proportional hazards model. The predictive ability of the models was assessed with McFadden R^2^ (explained variance) and c-statistics (discrimination).

**Results:**

A total of 3785 trauma patients (39% of total study population) responded to at least one follow-up questionnaire. Mean medical costs per patient (€9,710) and mean productivity costs per patient (€9,000) varied widely. Prognostic factors for high medical costs were higher age, female gender, spine injury, lower extremity injury, severe head injury, high injury severity, comorbidities, and pre-injury health status. Productivity costs were highest in males, and in patients with spinal cord injury, high injury severity, longer length of stay at the hospital and patients admitted to the ICU. Prognostic factors for RTW were high educational level, male gender, low injury severity, shorter length of stay at the hospital and absence of comorbidity.

**Conclusions:**

Productivity costs and RTW should be considered when assessing the economic impact of injury in addition to medical costs. Prognostic factors may assist in identifying high cost groups with potentially modifiable factors for targeted preventive interventions, hence reducing costs and increasing RTW rates.

## Introduction

Trauma is considered an important public health problem. Almost 80,000 patients (47 patients per 10,000 inhabitants) were admitted to a hospital due to an injury in 2017 in the Netherlands [1]. Furthermore, trauma is a major cause of death, and both short- and long-term disability in young adults [2].

The economic burden of injury consists of both medical and productivity costs. Medical costs are rising the last decades, making it an important societal and political topic [3]. These high costs are mainly due to the high number of (minor) injuries and the increasing costs of health care [4]. Productivity costs are based on the period of absence from work. Although most individuals with minor injuries rapidly recover and return to their daily activities, a significant part of the patients suffer long-term disabilities resulting in a long period of absenteeism at work [5].

Previous research on the economic burden of injury has focused mainly on specific injuries or age groups [6–9]. One study focused on health care costs and productivity costs of both minor and severe injuries [10]. Risk groups were identified, based on external cause, injury groupings, age and sex. They concluded that elderly females with hip fracture, young men with traffic injury or soccer injuries and bicycle or motorcycle injuries among all ages are known risk groups for high costs [10]. Previous research on return to work (RTW) provided prognostic factors for patients with traumatic brain injury (TBI)[11,12], patients with work-related injuries [13,14], major trauma [15] and extremity injury [16] and included, among others, the following prognostic factors: age, multiple injuries, injury severity and gender.

The prediction of costs and RTW after injury can enable policymakers to prioritize prevention and quality of care improvement, based on patient characteristics, pre-injury status and comorbidities of the patients. Prevention, intervention strategies and medical practice can target costly patients to reduce the economic burden. To our knowledge, no prediction models for medical costs, productivity costs and RTW in the total clinical trauma population have been developed.

The aim of this study was to determine prognostic factors for medical costs, productivity costs and RTW during the first two years after trauma in the clinical trauma population.

## Methods

### Study design

This study was performed with data from the Brabant Injury Outcome Surveillance (BIOS) study [17]. The BIOS study is a prospective observational follow-up cohort study that was approved by the Medical Ethics Committee Brabant (NL50258.028.14) and was registered at ClinicalTrials.gov (NTC02508675). The BIOS study enrolled all adult (≥18 years) trauma patients admitted to a ward or ICU in the region Noord-Brabant, the Netherlands, from August 2015 through November 2016 because of an injury, regardless of the injury severity. Exclusion criteria were insufficient knowledge of the Dutch language, no place of residence, or hospital admission due to pathological fractures.

Questionnaires were completed by a proxy if patients were incapable of completing the self-reported questionnaires. All participants or proxy informants signed informed consent. Patients or proxy informants were asked to complete the self-reported questionnaires at 1 week, 1, 3, 6, 12 and 24 months after injury.

### Outcome measures

Outcome measures were medical costs (in-hospital and post-hospital costs), productivity costs and RTW.

In-hospital costs involved the treatments and all activities during admission (i.e. emergency department visit, diagnostics, admission to ICU and ward and transport to hospital). In-hospital activities were registered after trauma and were obtained from the trauma registry and hospital registries.

The self-reported questionnaires at 1, 3, 6, 12 and 24 months after injury included the Institute for Medical Technology Assessment (iMTA) Medical Consumption Questionnaire (iMCQ) [18]. The iMCQ is a non-disease specific questionnaire for measuring post-hospital costs. Patients reported the number of appointments with medical specialists, whether they received home care, and stay or treatment at a medical facility. The questionnaires at 12 and 24 months after injury informed on homecare, GP consult, company doctor consult, psychologist and physiotherapist visits only.

Unit costs of health care activities were retrieved from a cost-reference manual [19]. Costs of diagnostics were based on unit costs from hospital price lists, the Dutch health care authority (NZa) and previous research [20–27]. Health care use was multiplied with the costs per unit. In-hospital costs and post-hospital costs were calculated by multiplying all activities with the corresponding unit price [28].

The self-reported questionnaires also included the iMTA Productivity cost questionnaire (iPCQ)[29,30] to assess RTW and to facilitate calculation of costs concerning productivity loss. Patients were asked about (first) RTW and the period of absenteeism. The costs of productivity loss were calculated with the friction cost method. This method estimates the costs of productivity loss based on an average individual earning of a certain friction period; theoretical time until another unemployed person replaced the individual who is absent. In line with previous research the friction period was set at 85 working days [19]. If working hours of patients were missing, these missing values were replaced with the national mean specified for sex. According to Statistics Netherlands (CBS) (2019) [31] the mean working hours for men were 36 hours per week and the mean working hours for women were 26 hours per week. Productivity loss was calculated by multiplying the missed working hours with the mean Dutch hourly wage rate, also specified for sex [19]. Productivity costs were calculated for the working age population (18–67 years). For the calculation of total mean costs (medical + productivity costs), productivity costs for patients aged >67 and patients without paid employment were equal to 0.

### Prognostic factors

#### Patient characteristic

Possible patient-related prognostic factors for all outcome measures were gender, age, educational level, pre-injury frailty, living situation and pre-injury health status which were collected from the questionnaires. Educational level was categorized in low (primary education or preparatory secondary vocational education, or no diploma), middle (university preparatory education, senior general secondary education or senior secondary vocational education and training), and high (university of applied science or an academic degree). Pre-injury frailty was measured with the Groningen Frailty Index (GFI) [32] at 1 week or 1 month (if patients did not participate at 1 week after injury) in patients aged ≥65. A sum score of 4 or higher for the GFI was considered frail and patients under 65 years were considered not frail. Pre-injury health status was measured with the EQ-5D-3L 1 week or 1 month (if patients did not participate at 1 week after injury). A utility score was calculated by using the Dutch tariffs [33].

#### Clinical variables

Clinical variables and injury characteristics were collected with the Brabant Trauma Registry (BTR). Data from the BTR was linked with data from the BIOS-study. Possible clinical prognostic factors for medical costs, productivity costs and RTW were the presence of comorbidities, cause of injury (7 categories: home/leisure, traffic, occupational, sport, self-harm, violence or other), Injury Severity Score (ISS), the functional capacity index (FCI), injury classification and Length of Stay (LOS). The FCI and ISS were based on the Abbreviated Injury Scale (AIS) codes (AIS-90, update 2008) [34].

AIS was used to create injury group classifications representing the most common types of injuries. In total, 14 injury groups were created: 3 lower extremity (pelvic injury, hip fracture, and tibia fracture / complex foot fracture or distal/shaft femur fracture), 2 upper extremity (shoulder and upper arm injury, and radius, ulna or hand fracture), 2 head (AIS-head≤2, and AIS-head≥3), 1 face, 2 Thorax (thorax injury, and rib fracture), 2 abdomen (AIS-abdomen≤2, and AIS-abdomen≥3) and 2 spine (spinal cord injury or brachial plexus lesion, and stable vertebral fracture or disc injury) injury groups. Patients who suffered multiple injuries were classified in multiple injury group classifications. LOS was not considered as prognostic factor for in-hospital costs because LOS was directly used to calculate medical costs.

### Statistical analysis

Results are reported according to the TRIPOD guidelines [35]. Analyses were performed in SPSS V.24 (statistical package for social sciences, Chicago, Illinois, USA) and R version 3.4.2 (R foundational for statistical computing, Vienna, Austria).

Patient characteristics were compared between responders and non-responders, with Mann-Whitney U tests and Chi-square tests for continuous and categorical variables respectively. Mean, median and interquartile range (IQR) of total costs, medical costs and productivity costs were calculated.

Missing baseline characteristics were imputed according to multiple imputation by using the Multivariate Imputations by Chained Equations (MICE) procedure with 15 imputations and 5 iterations [36]. The imputation model included baseline characteristics, injury characteristics and summary scores of the follow-up questionnaires. Missing outcome values were excluded from analyses (n = 264 for medical costs).

Prognostic factors for medical costs and productivity costs were assessed with log-linked gamma generalized linear models (GLM). The predictive ability was measured with explained variance (McFadden pseudo-R^2^). The relative difference in mean costs (exp[parameter estimate]) with 95% Confidence Interval (CI) were reported for the three models.

Prognostic factors for RTW were assessed with a Cox proportional hazards model. The analyses for RTW and productivity costs included patients aged ≤67 years who had paid employment prior to injury (n = 1236). The period of absenteeism (weeks) was set as time variable and RTW (1 = RTW, 0 = no RTW) as the dependent variable. The proportional hazards assumption (the ratio of the hazards for patients was constant over time) was checked visually with Kaplan-Meier curves. Hazard Ratios (HR) and 95% CI were reported. The predictive ability of the model was assessed with the C-statistic (a measure of goodness of fit for binary outcomes). A p-value of <0.05 was considered statistically significant.

## Results

### Patient and study characteristics

A total of 3785 trauma patients (39% of total study population, n = 9774) completed at least one follow-up questionnaire on health care use and RTW in the context of the BIOS-study **(S1 Fig)**. Responders had a mean age (SD) of 64.2 (18.9) years and 1911 (50.5%) were female **(Table 1)**. The median ISS (IQR) was 5 (4–9) and the median (IQR) length of stay at the hospital was 4 (2–8) days.

**Table 1:** Patient characteristics for the research population; Responders and non-responders.

	Responders to BIOS^a^	Non-responders	p-value*
***n***	3785	5989	
**Mean age (SD)**	64.2 (18.9)	64.4 (22.5)	0.529
**Females (n)**	1911 (50.5%)	3127 (52.2%)	0.097
**Median ISS (IQR)**	5 (4–9)	5 (2–9)^b^	<0.001
**Mean ISS (SD)**	6.6 (5.0)	6.2 (4.7)^b^	
**Median LOS (IQR)**	4 (2–8)	4 (2–8)^c^	0.537
**Admission to ICU (n)**	284 (7.5%)	366 (6.1%)	<0.01
**Injury classification (n)**			
Pelvic injury	250 (6.6%)	194 (6.5%)	
Hip fracture	979 (25.9%)	1386 (23.1%)	
Tibia, complex foot or femur fracture	443 (11.7%)	631 (10.5%)	
Shoulder and upper arm injury	354 (9.4%)	536 (8.9%)	
Radius, ulna or hand fracture	243 (6.4%)	348 (5.8%)	
Head injury with AIS ≤ 2	1013 (26.8%)	1754 (29.3%)	
Head injury with AIS ≥ 3	143 (3.8%)	224 (3.7%)	
Facial injury	196 (5.2%)	356 (5.9%)	
Thoracic injury	161 (4.3%)	199 (3.3%)	
Rib fracture	421 (11.1%)	518 (8.6%)	
Abdominal injury AIS ≤ 2	74 (2.0%)	102 (1.7%)	
Abdominal injury AIS ≥ 3	29 (0.8%)	37 (0.6%)	
Spinal cord injury	18 (0.5%)	19 (0.3%)	
Stable vertebral fracture or disc injury	238 (6.3%)	312 (5.2%)	

^a^missing items for responders to the BIOS questionnaires were imputed.

^b^missing values: 406

^c^missing values: 456

*Student’s t-test with unequal variance for age, Mann-Whitney U tests for ISS and LOS and Chi-square tests for gender and admission to ICU.

Abbreviations: AIS, Abbreviated Injury Scale; ICU, Intensive Care Unit; IQR, Inter Quartile Range; ISS, Injury Severity Score; n, number; SD, Standard Deviation

Responders (n = 3785) were more often admitted to the intensive care unit (ICU), and were more severely injured, according to the ISS (median [IQR] 5 [4–9] and 5 [2–9] respectively) compared to the non-responders (n = 5989).

### Costs of trauma

**Fig 1:**
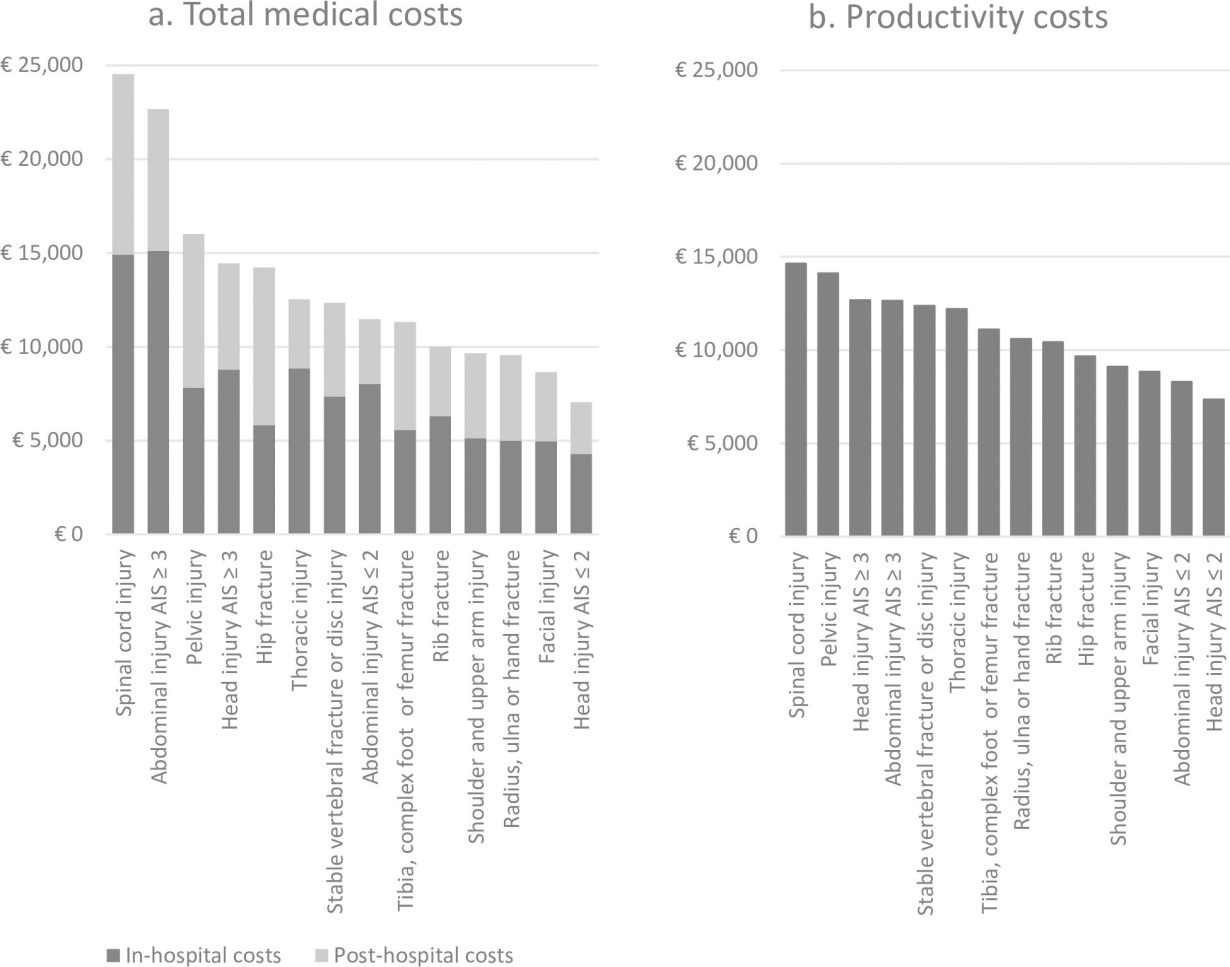
Mean medical (a) and productivity costs (b) in euros for injury classifications ordered from high to low.

Mean total costs per respondent were €12,970 (median: €7,290, IQR: €4,010-€15,960) (**Table 2**), of which medical costs comprised 75% and productivity costs comprised 25%. Highest mean total costs and medical costs per patient based on injury classifications were found in patients with spinal cord injury (mean: €36,720, 67% medical costs and 33% productivity costs [median €20,690, IQR: €10,200-€69,070]), followed by patients with severe abdominal injury (mean: €31,540, 72% medical costs and 28% productivity costs [median €18,200, IQR: €9,040-€44,390]) **(Fig 1A)**. Patients with ISS>15 also showed high mean medical and high mean productivity costs per respondent (€24,380 and €18,770 respectively).

**Table 2:** Mean and median (IQR) total costs in euros (€) of medical and productivity loss costs in the clinical trauma in population in the first two year after trauma.

	Total costs^c^ (€)		Medical costs (€)		Productivity loss costs^a^ (€)	
	N = 3172		N = 3521		N = 1032	
	Mean	Median (IQR)	Mean	Median (IQR)	Mean	Median (IQR)
**Total study population**	12970	7290 (4010–15960)	9710	4900 (2780–9300)	9000	7410 (2770–14900)
**Age (years)**						
18–44	13560	9290 (4460–18620)	6040	2800 (1910–5450)	8630	6880 (2640–14970)
45–64	14110	9820 (4880–19600)	7160	4000 (2480–7100)	9280	7750 (3300–14970)
65–74^b^	9020	5060 (3120–9060)	8780	4980 (3060–8730)	7130	3770 (2120–10080)
≥75	14140	7090 (4120–15590)	14140	7090 (4120–15590)		
**Gender**						
Male	12900	7180 (3860–18540)	8090	4070 (2380–7740)	10480	9660 (2940–16760)
Female	13020	7360 (4130–14520)	11310	5700 (3320–11150)	6490	6160 (2640–10080)
**Educational level**						
Low	13090	7210 (3980–15490)	11020	5650 (3220–11510)	10590	9880 (4500–16760)
Middle	12840	7440 (3980–16800)	8010	4140 (2390–7510)	9590	8620 (3090–16760)
High	12780	7300 (4070–15180)	8570	4050 (2460–7410)	6900	5200 (2150–10850)
**Injury classifications**						
Pelvic injury	19620	11620 (6340–16740)	16010	8820 (4750–17570)	14110	14900 (9360–18620)
Hip fracture	15400	8200 (4850–18130)	14230	6920 (4360–14460)	9670	9170 (5440–14970)
Tibia, complex foot or femur fracture	15870	10220 (5220–21490)	11330	5860 (3610–11210)	11100	10080 (4470–16760)
Shoulder and upper arm injury	12870	8630 (4980–16540)	9660	5700 (3130–9330)	9110	7750 (3520–14180)
Radius, ulna or hand fracture	14560	8780 (4530–20400)	9550	4840 (2600–10090)	10590	9300 (3740–16760)
Head injury AIS ≤ 2	9970	5230 (3010–11990)	7060	3430 (2180–6700)	7340	5240 (2040–12440)
Head injury AIS ≥ 3	17620	8650 (5440–21500)	14450	6660 (4020–15420)	12680	13930 (6100–17180)
Facial injury	13260	6590 (3270–15100)	8660	3530 (2260–7980)	8830	6200 (2650–12400)
Thoracic injury	18450	13600 (6500–22610)	12520	6820 (4100–11960)	12200	10970 (5990–16760)
Rib fracture	14570	8670 (4730–17770)	10020	5400 (3150–9260)	10420	9240 (4230–16760)
Abdominal injury AIS ≤ 2	15810	8430 (4010–15870)	11480	4970 (2690–13020)	8290	6880 (2730–12460)
Abdominal injury AIS ≥ 3	31540	18200 (9040–44390)	22660	10080 (5730–33460)	12640	10840 (5910–18620)
Spinal cord injury	36720	20690 (10200–69070)	24540	11870 (5460–32570)	14640	16760 (7750–18620)
Stable vertebral fracture or disc injury	18110	12460 (5710–22360)	12350	6240 (3430–11730)	12370	12400 (7450–16760)
**ISS**						
1–3	7830	4360 (2610–8560)	5160	2670 (1900–4720)	5590	2950 (1550–8370)
4–8	11980	7380 (3970–15660)	7870	4210 (2570–7760)	9650	8510 (3490–15510)
9–15	15090	8570 (4950–18560)	12670	6580 (4130–12090)	10610	9900 (5450–16760)
>15	24380	16660 (8220–32910)	18770	9780 (5830–22380)	13460	13940 (8860–18620)
**External cause**						
Home and leisure	13230	7280 (4020–15130)	11300	5610 (3290–11250)	8780	7580 (3050–13860)
Traffic	11930	6910 (3900–15400)	8090	4270 (2440–7740)	8210	6600 (2650–13100)
Occupational	20370	19920 (4730–27240)	7830	3450 (2100–7600)	14640	7450 (16760–16760)
Sport	10980	7840 (4130–16020)	4500	3090 (2190–5020)	8320	6840 (2660–13530)
Self-harm	11830	9290 (3170–20770)	7850	6680 (3150–13100)	7600	7000 (1340–14430)
Violence	10460	5240 (3990–9240)	7140	3250 (1530–5410)	7880	5070 (1980–12470)
Other	11500	6110 (3570–18270)	7900	3710 (1950–11740)	6470	4980 (1160–12450)

^a^Productivity loss costs were only assessed for patients aged 18–67 years with paid employment before injury (N = 1236, 204 missing productivity loss costs).

^b^Category changed to 65–67 for productivity loss costs.

^c^Total costs were only calculated for patients with both known medical costs and productivity loss costs.

Abbreviations: AIS, Abbreviated Injury Scale; CI, Confidence Interval; HS, Health Status; ISS, Injury Severity Score; LOS, Length of Stay; NA, Not Applicable.

Mean medical costs per patient were €9,710 (median: €4,900, IQR: €2,780-€9,300). All variables showed significant associations with medical costs in the univariable GLM **(S1 Table)**. Higher age was independently associated with increased medical costs in the multivariable GLM **(Table 3)**. Medical costs were on average 1.77 (95% CI: 1.48, 2.12) times higher in patients aged ≥75 compared to patients aged 18–44. Pelvic injury, tibia, complex foot or femur fracture, severe head injury, severe abdominal injury, spinal cord injury or stable vertebral fracture/disc injury were associated with increased medical costs. Besides, female gender, higher ISS and ≥2 comorbidities were prognostic factors for higher medical costs compared to male gender, low ISS and no comorbidities respectively. McFadden R^2^ of the multivariable model was 31,5%.

**Table 3:** The relative difference with multivariable generalized linear models for medical costs and productivity costs and multivariable cox proportional hazards model for RTW in the first two years after trauma.

	Generalized linear models		Cox proportional hazards model
	Medical costs	Productivity costs	RTW
	N = 3109^d^	N = 939^e^^,^^f^	N = 1015^e^^,g^
	Exp(E) (95% CI)	Exp(E) (95% CI)	Hazard Ratio (95% CI)
**Age (years)**			
18–44	Ref	Ref	Ref
45–64	1.16 (1.00, 1.34)	0.96 (0.86, 1.07)	1.08 (0.92, 1.26)
65–74^b^	1.25 (1.05, 1.48)	0.62 (0.45, 0.87)	1.48 (0.88, 2.49)
≥75	1.77 (1.48, 2.12)	NA	NA
**Female gender**	1.13 (1.03, 1.25)	0.68 (0.62, 0.76)	0.82 (0.71, 0.95)
**Educational level**			
Low^a^	Ref	Ref	Ref
Middle	0.92 (0.82, 1.03)	0.93 (0.82, 1.05)	1.27 (1.06, 1.53)
High	1.07 (0.94, 1.21)	0.71 (0.62, 0.80)	2.10 (1.74, 2.55)
**Injury classifications**			
Pelvic injury	2.01 (1.66, 2.44)	1.18 (0.95, 1.46)	0.84 (0.61, 1.15)
Hip fracture	1.19 (0.99, 1.44)	1.02 (0.82, 1.27)	0.87 (0.63, 1.20)
Tibia, complex foot or femur fracture	1.58 (1.34, 1.86)	1.14 (0.98, 1.33)	0.70 (0.56, 0.88)
Shoulder and upper arm injury	1.12 (0.95, 1.32)	1.00 (0.86, 1.17)	0.98 (0.78, 1.23)
Radius. ulna or hand fracture	1.00 (0.83, 1.21)	1.14 (0.96, 1.36)	0.80 (0.62, 1.02)
Head injury with AIS ≤ 2	0.86 (0.76, 0.97)	0.87 (0.77, 0.98)	1.04 (0.88, 1.23)
Head injury with AIS ≥ 3	1.58 (1.18, 2.12)	1.06 (0.78, 1.46)	0.87 (0.55, 1.39)
Facial injury	1.12 (0.91, 1.38)	0.92 (0.75, 1.11)	0.92 (0.70, 1.22)
Thoracic injury	1.07 (0.83, 1.38)	0.93 (0.74, 1.17)	1.09 (0.78, 1.53)
Rib fracture	1.02 (0.86, 1.21)	0.99 (0.84, 1.17)	1.20 (0.94, 1.51)
Abdominal injury AIS ≤ 2	0.93 (0.66, 1.32)	0.79 (0.59, 1.04)	1.62 (1.08, 2.42)
Abdominal injury AIS ≥ 3	1.81 (1.06, 3.09)	0.85 (0.58, 1.25)	1.54 (0.86, 2.78)
Spinal cord injury	2.62 (1.34, 5.11)	1.28 (0.68, 2.40)	0.66 (0.28, 1.59)
Stable vertebral fracture or disc injury	1.29 (1.06, 1.57)	1.39 (1.15, 1.67)	0.66 (0.50, 0.87)
**ISS**			
1–3	Ref	Ref	Ref
4–8	1.11 (0.96, 1.29)	1.36 (1.17, 1.57)	0.72 (0.59, 0.89)
9–15	1.50 (1.25, 1.80)	1.40 (1.15, 1.70)	0.62 (0.47, 0.82)
>15	1.89 (1.37, 2.60)	1.27 (0.91, 1.77)	0.67 (0.42, 1.07)
**Length of stay at hospital (days)**	NA^c^		
1–2		Ref	Ref
3–7		1.25 (1.10, 1.43)	0.74 (0.61, 0.89)
8–14		1.50 (1.24, 1.81)	0.53 (0.40, 0.70)
>14		1.54 (1.15, 2.08)	0.41 (0.26, 0.65)
**External cause**			
Home and leisure^a^	Ref	Ref	Ref
Traffic	0.97 (0.87, 1.09)	0.97 (0.86, 1.09)	1.03 (0.87, 1.23)
Occupational	1.08 (0.86, 1.36)	1.30 (1.08, 1.57)	0.79 (0.60, 1.04)
Sport	0.68 (0.56, 0.83)	0.91 (0.78, 1.06)	1.31 (1.06, 1.63)
Self-harm	0.96 (0.40, 2.32)	0.66 (0.31, 1.40)	0.89 (0.27, 2.89)
Violence	1.08 (0.69, 1.69)	0.95 (0.65, 1.40)	1.22 (0.69, 2.14)
Other	0.95 (0.58, 1.56)	0.88 (0.53, 1.45)	1.41 (0.69, 2.86)
**ICU admission**	NA^c^	1.27 (1.02, 1.58)	0.82 (0.59, 1.14)
**Number of comorbidities**			
0^a^	Ref	Ref	Ref
1	1.11 (0.98, 1.24)	0.97 (0.86, 1.09)	1.05 (0.88, 1.25)
≥2	1.35 (1.18, 1.54)	1.01 (0.86, 1.20)	0.92 (0.71, 1.18)
**Frail**	1.06 (0.91, 1.24)	NA^c^	NA^c^
**Pre-injury health status**	0.63 (0.49, 0.82)	0.90 (0.58, 1.40)	1.69 (0.86, 3.31)
**Intercept**	5102.99 (3761.04, 6923.74)	8370.42 (5342.99, 13113.25)	NA
**McFadden R**^**2**^	**31.50%**	**19.80%**	**NA**
**C-statistic (95% CI)**	**NA**	**NA**	**0.700 (0.682. 0.718)**

^a^Reference category of categorical variable.

^b^Category changed to 65–67 for productivity loss costs and RTW.

^c^variables were not considered as predictors.

^d^missing values: 23 Number of comorbidities, 389 Pre-injury health status, 264 medical costs.

^e^working population N = 1236.

^f^missing values: 3 Number of comorbidities, 90 Pre-injury health status, 204 missing productivity loss costs. ^f^missing values: 18 status RTW, 113 time to event, 87 Pre-injury health status, 3 Number of comorbidities.

Abbreviations: AIS, Abbreviated Injury Scale; CI, Confidence Interval; ISS, Injury Severity Score; NA, Not Applicable.

Average productivity costs per respondent were €9,000 (median: €7,410, IQR: €2,770-€14,900) within the working population. Highest mean productivity costs per patient were found in patients with spinal cord injury (€14,640, median: €16,760, IQR: €7,750-€18,620) and pelvic injury (€14,110, median: €14,900, IQR: €9,360-€18,620) **(Fig 1B)**. Mean productivity costs were also high in patients who were admitted due to occupational injury (mean €14,640 per patient) and in patients with ISS>15 (mean: €13,460 per patient).

Number of comorbidities, pre-injury health status and age showed not to be associated with productivity costs in the univariable GLM **(S1 Table)**. In contrast with the univariable GLM, lower age showed to be associated with higher productivity costs in the multivariable GLM. Prognostic factors for increased productivity costs were male gender, low educational level, stable vertebral fracture/disc injury, higher ISS, higher LOS, occupational injury and admission to the ICU in the multivariable GLM **(Table 3)**. McFadden R^2^ of the multivariable model was 19,8%.

### RTW

**Fig 2:**
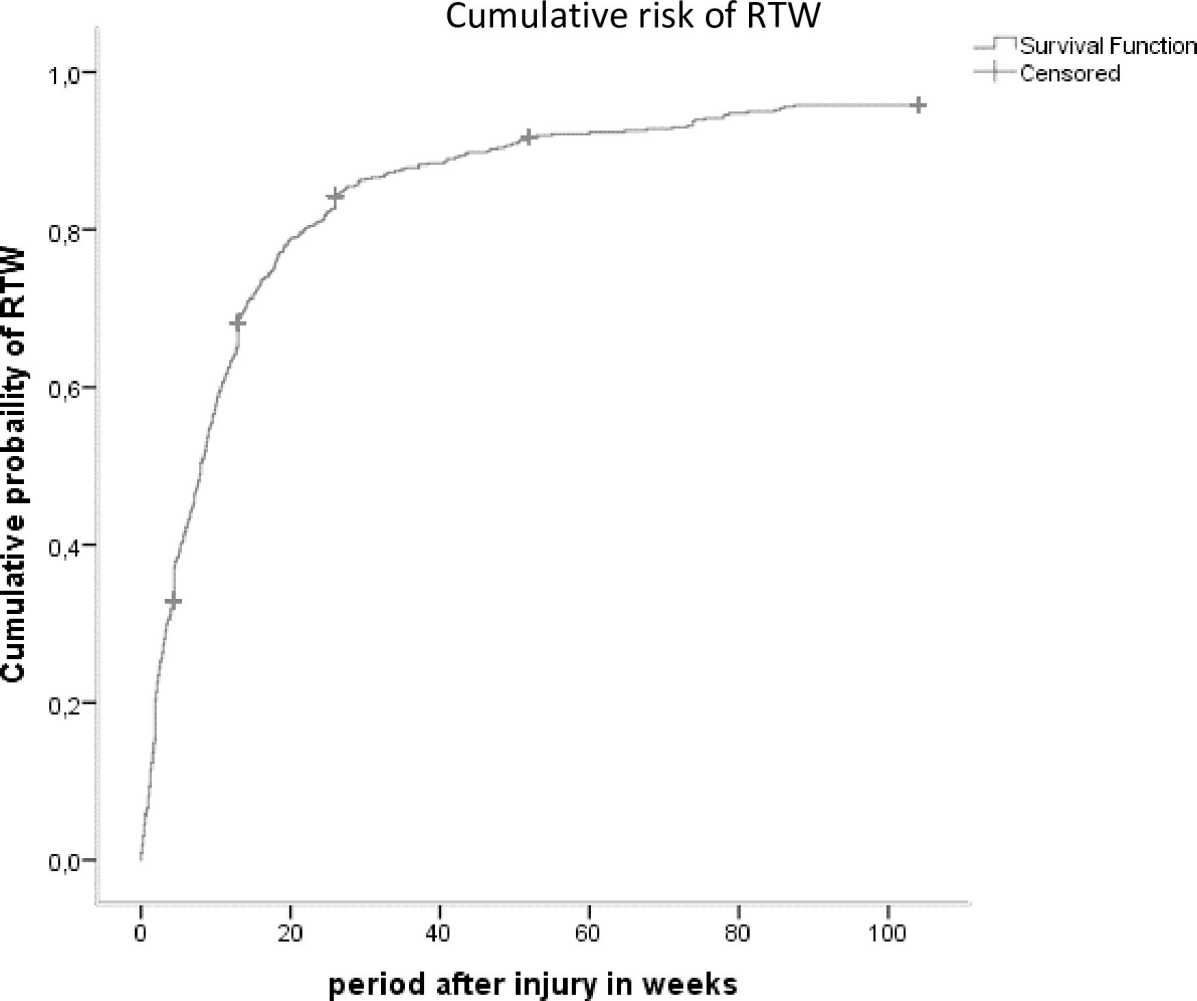
Rate of RTW after trauma in the working age study population during 24 months of follow-up (n = 1236).

Within the working age population (18–67 years, n = 1964) a total of 1,236 patients (63%) reported to have paid employment prior to injury, of which 131 patients (11%) had a missing value for time to event. For patients with known outcome (n = 1105), median survival weeks was 8 (IQR: 2.5–17.7). A total of 180 patients were censored due to loss to follow-up **(Fig 2)**. At 12 months after injury, 76% (n = 841) of the patients with known outcome returned to work and 6% (n = 69) did not return to work.

Age, gender, number of comorbidities and pre-injury health status showed no significant association with RTW in the univariable Cox regression analyses **(S1 Table)**. Prognostic factors for RTW in the multivariable analyses were higher age (HR: 1.48 [95% CI: 0.88, 2.49]), male gender (HR: 1.22 [95% CI: 1.05, 1.41]), higher educational level (HR: 1.27 [95% CI: 1.06, 1.53] for middle educational level and HR: 2.10 [95% CI: 1.74, 2.55] for high educational level compared to low educational level) and patients with a sport injury (HR: 1.31 [95% CI: 1.06, 1.63] compared to patients with home and leisure injury) **(Table 3)**. Injury classifications with decreased probability on RTW were tibia, complex foot or femur fracture (HR: 0.70 [95% CI: 0.56, 0.88]) and stable vertebral fracture/disc injury (HR: 0.66 [95% CI: 0.50, 0.87]). Besides, higher ISS and longer LOS showed a decreased probability on RTW. C-statistic of the multivariable Cox-regression model was 0.700 (95% CI: 0.682, 0.718).

## Discussion

This study explored prognostic factors for medical costs, productivity costs and RTW after trauma during 24 months of follow-up. A stable vertebral fracture or disc injury and higher ISS were independently associated with higher medical costs, higher productivity costs and longer period of absenteeism at work. Although female gender and higher age were prognostic factors for higher medical costs, they were also both associated with lower productivity costs after adjustment for confounding. A total of 5% of the patients with paid employment did not return to work at 12 months after injury. Important prognostic factors for RTW were higher educational level, higher age, low ISS and low LOS.

### Costs

In line with previous studies, higher age and female sex were associated with higher medical costs[10,37]. No clear trend was found between mean total costs per respondent and age, probably due to a different pattern in productivity costs among age categories. Other variables such as high ISS, specific body regions (abdomen, spine and brain injury) and LOS were also identified as prognostic factors for medical costs in a review[38].

Female sex is a prognostic factor for higher medical costs, but is also associated with lower productivity costs. The lower productivity costs might be explained by the fact that women more often work part-time compared to men in the Netherlands[39].

A Dutch study on the economic burden of injury reported the highest health care costs for hip fracture patients (€20,000 per patient)10. Although our study showed that hip fractures were in the top 5 of high medical costs (€14,230 per patient), several other injury classifications were considerably more expensive. However, the prevalence of hip fractures in the injury cohort is high, so this could have more impact on health care consumption and costs compared to the relatively low prevalence of more costly injuries (e.g. spinal cord injury or abdominal injury with AIS≥3).

### RTW

A previous study on RTW in major trauma patients also found comorbidities, pre-injury disability, and presence of spinal cord injury as prognostic factors for no RTW [40]. They also stated that older age was a prognostic factor for no RTW, in contrast to our results. This could be explained by differences in study population (major trauma vs all trauma).

Previous research found an association between unemployment and poor recovery [41,42]. This association is probably interchangeable; patients who experience for example pain or distress after trauma are more likely for no RTW and patients who are back at work soon after their injury, have a better recovery [43]. These factors can be influenced by tailored interventions. Future research should focus on these specific modifiable physical complaints and psychological distress after trauma and the medical costs and RTW. Interventions can help with the recovery, which could result in lower medical costs and sooner RTW.

The proportion of RTW in our study is higher compared to previous studies [44,45]. This could be explained by differences in characteristics of the studies; e.g. the injury severity in our study was lower [44] or differences in definition of RTW [45]. Our study showed that the probability of RTW decreased over time, but did not stabilize up to 24 months after injury. In contrast with RTW rates in moderate to severe TBI patients [46], in which RTW rates remained stable between 1 and 10 years after injury, RTW rates could still increase with time in our study population. A longer follow-up is necessary to conclude which patients remain unemployed.

### Strengths and limitations

A strength of this study is the large study population, including short-term (1 week) and long-term (two years). This study design allows to calculate costs and RTW in detail and could give an overview of costs and RTW in the total clinical trauma population. Furthermore, to our knowledge, this was the first attempt to develop prediction models for the total trauma population for both medical as productivity costs and also RTW. Productivity costs were based on the friction cost method. This method does not discriminate between patients who are 90 days absent at work or 2 years absent at work. Although there are no extra productivity costs, it does affect society and the patient. Therefore, a strength of this study is the addition of RTW (in days) up to 2 years after injury as outcome.

An important limitation in this study is that educational level is not taken into account for the calculation of productivity costs. Although different average wages were used for males and females, no distinction was made between average wages of the different educational levels. The analyses showed that high educational level was a prognostic factor for RTW, indicating that patients with high educational level were sooner back at work after trauma compared to patients with low educational level. However, because the wages per hour were similar to patients with low educational level, the significant result of high educational level as prognostic factor for lower productivity costs probably only indicates this shorter period of absenteeism at work instead of actually lower productivity costs. In addition, the proportion of patients with low educational level was higher in our study population, compared to the Dutch working population (29% vs 20%) [47]. This could have resulted in an overestimation of the total productivity loss costs. Future studies should assess this with average wages that are corrected for educational level.

In addition, costs due to presenteeism were not taken into account, resulting in a probable underestimation of the total productivity costs. Future research should take into account both absenteeism and presenteeism.

Another limitation of this study is the use of self-reported questionnaires which are prone to recall-bias. Patients were asked about care consumption and RTW over the period between the previous questionnaire and the current questionnaire. This period increased from 1 week for the first questionnaire to 12 months for the last follow-up questionnaire.

Next, the primary aim of the BIOS-study is to determine physical and psychological outcome after injury. This means that only survivors of an injury were included in the BIOS-study. Patients who did not survive until one week after trauma were excluded and costs were not calculated (n = 219, 2% of the BIOS-study population).

Furthermore, although the analyses were conducted in a large study population, only 39% of the total trauma population completed the necessary questionnaires. Patients who were fully recovered were probably more likely to be lost to follow-up. Differences between responders and non-responders indicate that more severely injured patients participated. This non-response bias could result in an overestimation of both medical and productivity costs.

Last, injury severity in our study population was probably low in contrast to other trauma populations. However, in the Dutch trauma registry, all patients who were admitted to the hospital are included, regardless of the ISS. In contrast with the 5% severely injured in the total Dutch trauma population, our cohort included more severely injured patients (7%)[1]. Compared to other countries, the number of severely injured patients is relatively low [48]. This could indicate that there are some limitations with the generalizability of this study. The Brabant region is considered representative for the total Dutch clinical trauma population. However, differences in trauma populations (e.g. ISS) and trauma care (e.g. prehospital care) could make international comparisons difficult.

### Implications

The findings of this study showed high medical costs, high productivity costs and long absenteeism from work. An intervention to help patients to earlier return to work after sustaining a trauma could reduce productivity costs and decrease the period of absenteeism at work. Considering the differences in prognostic factors for medical costs and productivity costs, it is important that medical costs and productivity costs are both taken into account and analysed separately. The identified prognostic factors in this study were all easy to detect patient and injury characteristics. This enables health practitioners and policy makers to inform patients and induce prevention interventions to reduce medical costs and productivity costs.

## Conclusion

Although many prognostic factors resulted in both higher medical costs and higher productivity costs, some factors showed differential effects. Productivity costs and RTW should be considered when assessing the economic impact of injury in addition to medical costs. Prognostic factors may assist in identifying high cost groups with potentially modifiable factors for targeted preventive interventions, hence reducing costs and increasing RTW rates.

## Supporting information

S1 Fig Flow diagram of study participation; the total number of unique patients that responded to the questionnaire on health care use and RTW was 3785. (PDF)

S1 Table The relative difference with univariable generalized linear models for medical costs and productivity costs and univariable cox proportional hazards model for RTW in the first two years after trauma. (DOCX)
