# Supplementary figures and images for: Prognostic factors for medical and productivity costs, and return to work after trauma
Source: PLoS One. 2020 Mar 25;15(3):e0230641. doi: 10.1371/journal.pone.0230641 (PMC7094860; doi:10.1371/journal.pone.0230641)

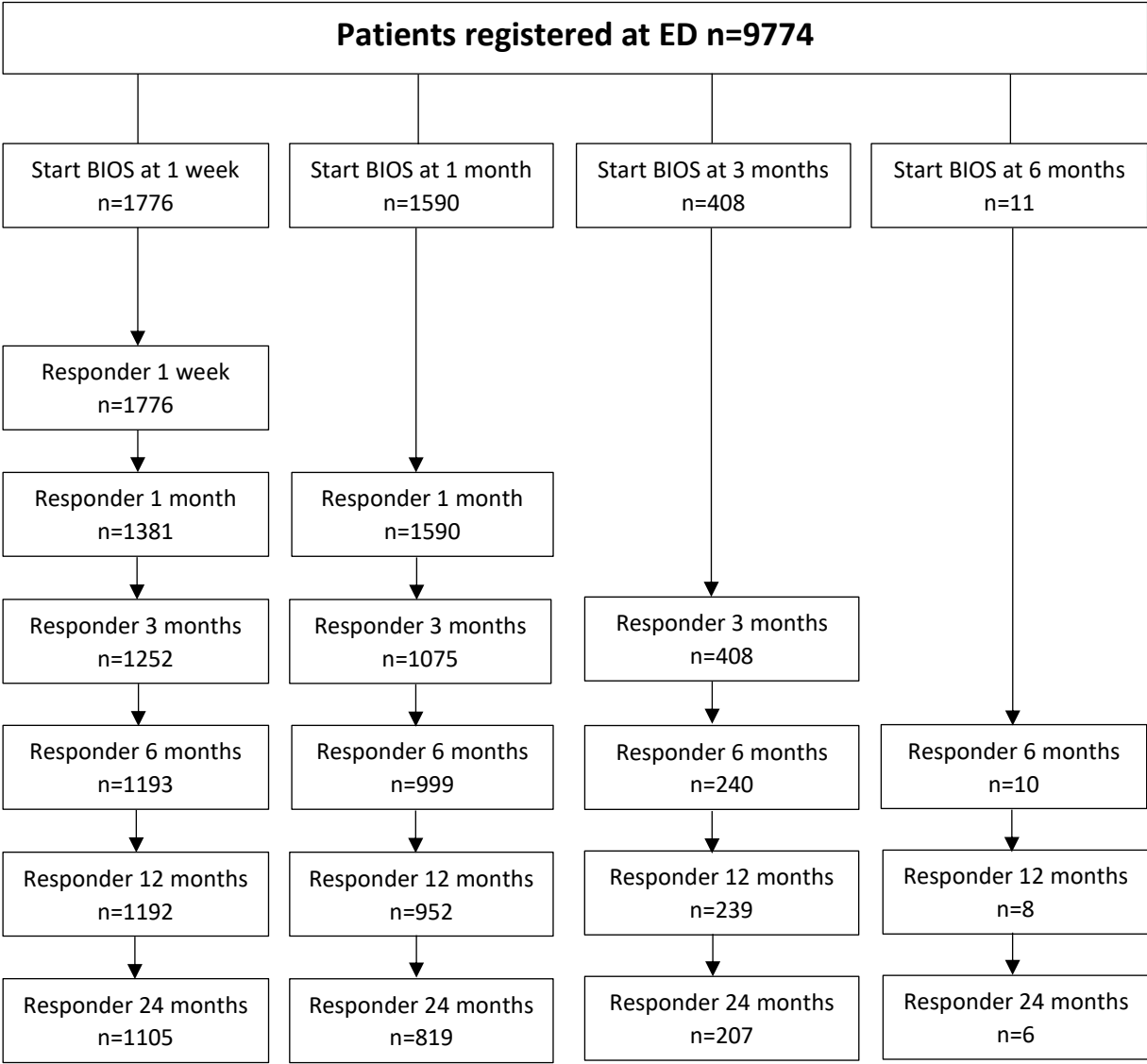

Supplement: S1 Fig — (PDF) [file pone.0230641.s001.pdf]
