# Supplementary material for: Prognostic factors for medical and productivity costs, and return to work after trauma
Source: PLoS One. 2020 Mar 25;15(3):e0230641. doi: 10.1371/journal.pone.0230641 (PMC7094860; doi:10.1371/journal.pone.0230641)
Supplement: S1 Table — (DOCX) [file pone.0230641.s002.docx]

**S2 Table. The relative difference with univariable generalized linear models for medical costs and productivity costs and univariable cox proportional hazards model for RTW in the first two years after trauma.**

|  | Generalized linear models | | Cox proportional hazards model |
| --- | --- | --- | --- |
|  | Medical costs  N=3521^d^ | Productivity costs  N=1236^e,f^ | RTW  N=1236^e,g^ |
|  | Exp(E) (95% CI) | Exp(E) (95% CI) | Hazard Ratio (95% CI) |
| Age (years)  18-44  45-64  65-74^b^  ≥75 | Ref  1.19 (1.01-1.39)  1.45 (1.22, 1.73)  2.34 (2.00, 2.74) | Ref  1.07 (0.97, 1.19)   - 1. 0.60, 1.13)   NA | Ref  0.93 (0.82, 1.07)  0.92 (0.57, 1.49)  NA |
| Female gender | 1.40 (1.26, 1.55) | 0.62 (0.57, 0.68) | 0.91 (0.80, 1.05) |
| Educational level  Low^a^  Middle  High | Ref  0.73 (0.64, 0.83)  0.78 (0.67, 0.90) | Ref  0.91 (0.81, 1.02)  0.65 (0.58, 0.74) | Ref  1.28 (1.09, 1.51)  2.05 (1.73, 2.42) |
| Injury classifications^f^ |  |  |  |
| Pelvic injury | 2.08 (1.70, 2.54) | 1.51 (1.24, 1.84) | 0.57 (0.43, 0.76) |
| Hip fracture | 2.31 (2.02, 2.64) | 1.20 (1.01, 1.44) | 0.65 (0.51, 0.83) |
| Tibia, complex foot or femur fracture | 1.60 (1.36, 1.89) | 1.39 (1.21, 1.60) | 0.56 (0.46, 0.68) |
| Shoulder and upper arm injury | 1.25 (1.05, 1.49) | 1.08 (0.93, 1.26) | 0.84 (0.68, 1.04) |
| Radius, ulna or hand fracture | 1.01 (0.83, 1.24) | 1.19 (1.00, 1.40) | 0.85 (0.67, 1.07) |
| Head injury AIS ≤ 2 | 0.82 (0.72, 0.93) | 0.79 (0.71, 0.89) | 1.14 (0.98, 1.33) |
| Head injury AIS ≥ 3 | 2.09 (1.61, 2.70) | 1.61 (1.25, 2.08) | 0.45 (0.31, 0.67) |
| Facial injury | 1.14 (0.91, 1.42) | 0.99 (0.82, 1.19) | 0.91 (0.70, 1.17) |
| Thoracic injury | 1.10 (0.83, 1.44) | 1.20 (0.97, 1.49) | 0.91 (0.67, 1.22) |
| Rib fracture | 1.09 (0.91, 1.30) | 1.11 (0.95, 1.30) | 1.03 (0.83, 1.29) |
| Abdominal injury AIS ≤ 2 | 0.92 (0.64, 1.32) | 0.75 (0.57, 0.98) | 1.43 (0.98, 2.09) |
| Abdominal injury AIS ≥ 3 | 1.56 (0.87, 2.80) | 1.08 (0.74, 1.57) | 1.02 (0.60, 1.74) |
| Spinal cord injury | 3.85 (1.92, 7.73) | 1.51 (0.85, 2.68) | 0.46 (0.20, 1.04) |
| Stable vertebral fracture or disc injury | 1.30 (1.06, 1.59) | 1.44 (1.22, 1.71) | 0.63 (0.49, 0.80) |
| ISS  1-3  4-8  9-15  >15 | Ref  1.53 (1.33, 1.75)  2.46 (2.15, 2.81)  3.64 (2.85, 4.66) | Ref  1.72 (1.53, 1.94)  1.89 (1.66, 2.16)  2.40 (1.95, 2.95) | Ref  0.54 (0.46, 0.63)  0.45 (0.37, 0.54)  0.33 (0.25, 0.45) |
| Length of stay at hospital (days)  1-2  3-7  8-14  >14 | NA^c^ | Ref  1.44 (1.29, 1.60)  1.98 (1.69, 2.31)  2.22 (1.72, 2.85) | Ref  0.60 (0.51, 0.69)  0.41 (0.33, 0.51)  0.27 (0.18, 0.39) |
| External cause  Home and leisure^a^  Traffic  Occupational  Sport  Self-harm  Violence  Other | Ref  0.72 (0.64, 0.81)  0.69 (0.54, 0.89)  0.40 (0.26, 1.87)  0.69 (0.26, 1.87)  0.63 (0.39, 1.04)  0.70 (0.39, 1.26) | Ref  0.93 (0.83, 1.03)  1.66 (1.40, 1.97)  0.95 (0.83, 1.10)  0.87 (0.42, 1.82)  0.89 (0.62, 1.27)  0.75 (0.45, 1.24) | Ref  1.09 (0.94, 1.27)  0.74 (0.58, 0.94)  1.43 (1.18, 1.73)  0.73 (0.24, 2.26)  1.39 (0.83, 2.33)  1.41 (0.72, 2.76) |
| ICU admission | NA^c^ | 1.58 (1.34, 1.87) | 0.61 (0.48, 0.77) |
| Number of comorbidities  0^a^  1  ≥2 | Ref  1.46 (1.29, 1.65)  2.02 (1.79, 2.28) | Ref  1.03 (0.92, 1.16)  1.08 (0.92, 1.26) | Ref  0.94 (0.80, 1.10)  0.84 (0.67, 1.04) |
| Frail | 1.67 (1.46, 1.92) | NA^c^ | NA^c^ |
| Pre-injury health status | 0.32 (0.25, 0.40) | 1.09 (0.71, 1.68) | 1.85 (0.99, 3.46) |

^a^Reference category of categorical variable, ^b^Category changed to 65-67 for productivity loss costs and RTW, ^c^variables were not considered as predictors. ^d^missing values: 23 Number of comorbidities, 389 Pre-injury health status, 264 medical costs. ^e^working population N=1236. ^f^missing values: 3 Nr of comorbidities, 90 Pre-injury health status, 204 missing productivity loss costs. ^f^missing values: 18 status RTW, 113 time to event, 87 Pre-injury health status, 3 Number of comorbidities.

*Abbreviations: AIS, Abbreviated Injury Scale; CI, Confidence Interval; ISS, Injury Severity Score; NA, Not Applicable.*
